# Aquatic Heteroptera (Nepomorpha, Gerromorpha) in small intermittent rivers of Ukraine steppe zone

**DOI:** 10.3897/zookeys.319.4328

**Published:** 2013-07-30

**Authors:** Maria A. Grandova

**Affiliations:** 1 UkrSCES, Frantsuzskiy Blvd., 89, Odessa, Ukraine

**Keywords:** Aquatic Heteroptera, Nepomorpha, Gerromorpha, steppe zone, small intermittent rivers

## Abstract

Small intermittent rivers are some of the most widespread types of water currents in the steppe zone. In the ecosystems of the intermittent rivers we have found 28 species of water bugs that compose the majority of the described fauna in the south Ukraine. Our study added two new species to the faunistic list of this zone (*Sigara fossarum*, *Hydrometra gracilenta)*, and finally confirmed the presence of *Micronecta scholtzi* in Ukraine. We also studied the seasonal changes of biotopic distribution and quantitative characteristics of aquatic Heteroptera. It was shown thatfor water bugs the ecosystem of the small intermittent rivers consists of three closely connected components: the riverbed, the flooded areas and the extra inundated constant basins. During the droughty period when the riverbed is dry, the extra inundated basins (including artificial ones – sandy pits) serve as refugia. The wintering of many species takes place there, especially when the riverbed is not filled before the cold period. However, the reproduction of most species takes place in the riverbed and associated flooded areas. Thus, this work is a confirmation and development of the concept for “a uniform architectonic complex of a river valley”.

## Introduction

The small intermittent rivers are some of the most widespread types of water currents in the south of Ukraine steppe zone. Under droughty climate conditions, they and the associated water bodies serve as refugia for aquatic organisms (Dyadichko 2008, 2009), while valleys serve as “ecological corridors” for the distribution of species from other landscape-climatic zones (Gramma 1974), playing a significant role in formation and maintenance of biodiversity in the steppe zone. The overall studying of small intermittent rivers is essential for understanding the function of the entire steppe biocenosis. Special studies of aquatic Hemiptera in this region haven’t been carried out yet.

## Materials and methods

**Figure 1.**
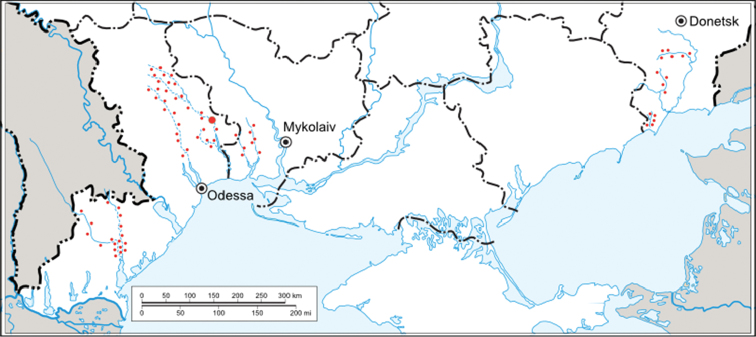
The studied area of Ukrainian steppe zone (sampling sites were marked with small red dots, the site of quantitative sampling – with a large red dot).

The materials of our own expeditions (2007-2011), the collections of V. Dyadichko, A. Martynov and V. Martynov, as well as the collection of the Zoology Department of Donetsk National University were examined in this study. During the study, small intermittent rivers and associated water bodies of Odessa, Nykolaev and Donetsk areas were examined. For the study of quantitative characteristics, the material from the middle Tiligul River and associated water bodies in the Berezovkij wildlife reserve was used (Fig. 1).

The following typical biotopes were studied:

1. The riverbed. The bottom is sandy or oozy and sandy, sometimes with stones. Depth up to 1,5 m, current speed up to 1 m/s. The vegetation is abundant including marsh (*Carex* spp., *Iris* spp., *Typha* spp., *Phragmites* spp.), and submerged forms (*Ceratophyllum* spp., *Utricularia* spp., *Chara* spp., *Ranunculus* spp., filamentous algae). At the end of May-June, the current decreases, then it turns into several small pools and by the end of July - the beginning of August completely dries up. In October-November or later, depending on precipitation, autumn filling of the riverbed occurs.
2. The flooded area can be divided into three zones: a) the water meadows with land cereals that are flooded at a high water level and dry at the end of April-May; b) the inundated lowlands which dry up after water meadows (at the end of May - the beginning of June), and where land and marsh vegetation are combined; c) inundated pools with an oozy bottom which dry last, and vegetation includes sedge, mosses and cane.
3. The constant extra inundated basins are not connected directly to the river, but they are filled with the river water filtering through the soil. They can be both natural and artificial (sandy, clay pits). The surface area is up to 200 m^2^, depth is up to 2-3 m, usually with a shallow coastal strip. Vegetation is poor. Some of these basins do not dry out even in the driest summer.

The periodization for the steppe intermittent rivers was offered by Dyadichko (2008). His division includes five periods, suitable also for aquatic Hemiptera:

1. **The early-spring period** continues from the ice melting and floods formation at the end of February – the beginning of March till the beginning of water vegetation growth at the end of March – April. Light day duration is about 10,9–12,3 h.
2. **The spring period** begins at the end of March, when water vegetation starts growing. It lasts till the depression of the water level in the flooded areas and the beginning of *Phragmites* spp. and *Typha* spp. growth at the end of April. Light day duration is about 12,4–14,2 h.
3. **The late-spring period** lasts through the end of April and May. It is characterized by desiccation of the flooded areas, normal water level in the riverbed, vegetation of *Phragmites* spp. and *Typha* spp., and flowering of iris marsh. Light day duration is about 14,3–15,4 h.
4. **The summer-autumn period** continues from June till the beginning of October. It is characterized by desiccation of the riverbed and many small basins. In June, the current is practically absent, water temperature is 24–27°C, and the semi-shipped vegetation (*Carex* spp., *Typha* spp., *Phragmites* spp.) and filamentous algae grow. Usually up to the end of July the riverbed has completely dried.
5. **The autumn-winter period** lasts from the filling of the riverbed in October – November till water freezing in December – the middle of February.

Quantitative samples were taken approximately every two weeks with the help of a Balfour-Browne hand net or hydrobiological drag, and also using meiobenthic methods for Micronectidae and nymphs of younger stages. Modified fish-traps and light attraction were also used for general collecting. In total, about 10000 specimens of aquatic Hemiptera were studied. They were identified based on the works of Kanyukova (2006), Savage (1989), Poisson (1957) and Wroblewski (1958). The systematic order is after Aukema and Rieger (1995) and Nieser (2002). The numbers and biomass of aquatic bugs from the quantitative samples were calculated per square meter.

## Results and discussion

In the ecosystems of the intermittent rivers, we have found 28 species of water bugs from 9 families of Nepomorpha: Corixidae – 12 (Corixinae – 11, Cymatiinae – 1), Micronectidae – 1, Naucoridae - 1, Nepidae – 2, Notonectidae – 2, Pleidae – 1, and Gerromorpha: Gerridae – 6, Hebridae – 1, Hydrometridae – 1, Vellidae – 1 (Table 1). This is the majority (67%) of the known fauna for the steppe zone in the south of Ukraine (Putshkov and Putshkov 1996). Two new species are added to the faunistic list of this zone (*Sigara fossarum*, *Hydrometra gracilenta*) and new data on biology and presence of recently confirmed in Ukraine *Micronecta scholtzi* (Grandova and Prokin 2012) are added. It has been found only in intermittent rivers and small steppe basins.

**Table 1.** List of species and occurrence of water bugs in small intermittent rivers and associated water bodies.

**Family**	**Species**	**Occurrence index (%)**
Nepidae	*Nepa cinerea* Linnaeus, 1758	3,92
	*Ranatra linearis* (Linnaeus, 1758)	1,96
Corixidae	*Corixa affinis* Leach, 1817	3,92
	*Corixa dentipes* Thomson, 1869	15,69
	*Cymatia rogenhoferi* (Fieber, 1864)	11,76
	*Hesperocorixa linnaei* (Fieber, 1848)	74,51
	*Paracorixa concinna* (Fieber, 1848)	9,80
	*Sigara assimilis* (Fieber, 1848)	13,73
	*Sigara fossarum* (Leach, 1817)	1,96
	*Sigara iactans* Jansson, 1983	9,80
	*Sigara lateralis* (Leach, 1817)	78,43
	*Sigara nigrolineata* (Fieber, 1848)	7,84
	*Sigara stagnalis* (Leach, 1817)	64,71
	*Sigara striata* (Linnaeus, 1758)	66,67
Micronectidae	*Micronecta scholtzi* (Fieber, I860)	19,61
Naucoridae	*Ilyocoris cimicoides* (Linnaeus, 1758)	21,57
Notonectidae	*Notonecta glauca* Linnaeus, 1758	27,45
	*Notonecta viridis* Delcourt, 1909	29,41
Pleidae	*Plea minutissima* Leach, 1817	29,41
Hebridae	*Hebrus ruficeps* Thomson, 1871	1,96
Hydrometridae	*Hydrometra gracilenta* Horvath, 1899	1,96
Veliidae	*Microvelia reticulata* (Burmeister, 1835)	5,88
Gerridae	*Gerris argentatus* Schummel, 1832	25,49
	*Gerris asper* (Fieber, 1860)	3,92
	*Gerris lacustris* (Linnaeus, 1758)	1,96
	*Gerris odontogaster* (Zetterstedt, 1828)	13,73
	*Gerris thoracicus* Schummel, 1832	23,53

*Sigara stagnalis*, *Sigara striata*, *Sigara lateralis*,*Hesperocorixa linnaei* belong to dominants, *Notonecta glauca*, *Notonecta viridis*,*Plea minutissima*, *Gerris argentatus*, *Gerris thoracicus*, *Ilycoris cimicoides* – to subdominants.

**Figure 2.**
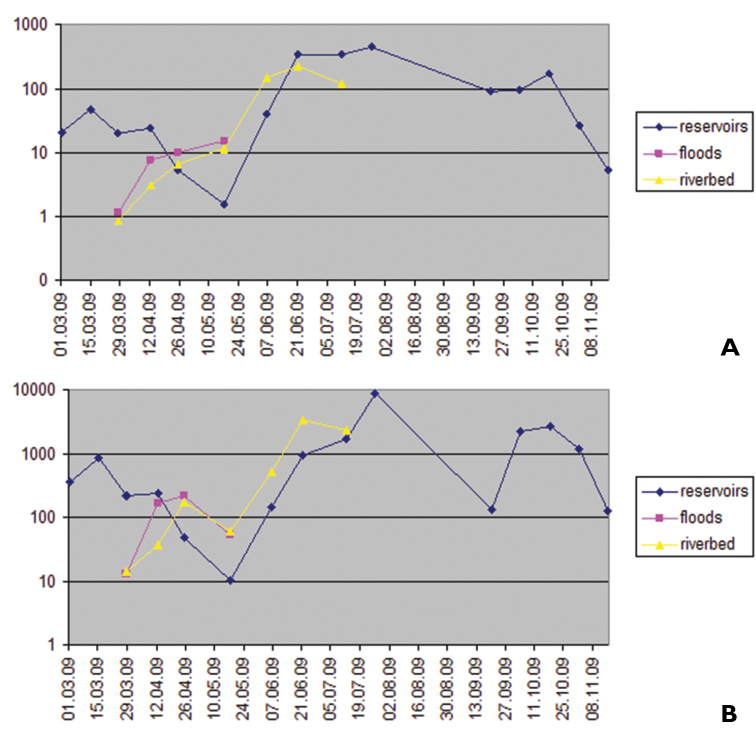
Seasonal changes in numbers (**A** ind./m^2^) and biomass (**B** mg/m^2^) of water bugs in Tiligul River and associated water bodies.

The results of quantitative characteristics of water bugs are shown on Fig. 2. Data on numbers and biomass of Micronectidae species will be discussed separately.

During **the early-spring period**, the specific structure and quantitative characteristics of water bugs in different biotopes are defined by weather conditions during the year, the period of previous autumn filling of the riverbed playing a very important role.

During the autumn of 2007, the riverbed was filled in October, therefore a portion of the water bugs remained there for wintering. Next year the first specimens, which belonged to *Sigara stagnalis*, were caught in the riverbed in the middle of February, soon after ice melting. At the beginning of March, water bugs were observed both in the riverbed and in the flooded areas, and *Sigara striata*, *Sigara stagnalis*, *Sigara lateralis*, *Hesperocorixa linnaei* prevailed.

In 2008 the autumn filling of the riverbed occurred late, therefore the water bugs did not winter there and during the next early-spring period were absent in the quantitative samples from the riverbed. During general collecting we found singular specimens of Gerridae. The highest numbers and biomass were observed in extra inundated basins (sandy pits) where the majority of Nepomorpha wintered. The highest numbers during this period belonged to Corixidae. The maximum of biomass at the beginning of this period also belonged to Corixidae, but from the beginning of March Notonectidae started to dominate by biomass. In 2009 the numbers at this period reached 48,15 ind./m^2^, the biomass – 871 mg/m^2^. The highest numbers belonged to *Sigara lateralis*, the highest biomass - to *Notonecta glauca*.

In the early-spring period the overwintered nymphs of Micronectidae gather near the shore line, and their numbers can reach high values. At the beginning of March 2009, their numbers were 0,37 ind./m^2^, and the biomass – 0,19 mg/m^2^

During **the spring period,** water bugs migrated to the flooded areas and the riverbed. The area of suitable habitats sharply increased and as a result, the number and the biomass of water bugs per square meter decreased. At the beginning of the spring period, the majority of Nepomorpha continued to remain in the extra inundated basins, therefore the numbers and the biomass of water bugs there exceeded these values both in the riverbed and in the flooded area.

At the end of March 2009, the numbers of water bugs in the riverbed were 0,83 ind./m^2^, the biomass was 14,17 mg/m^2^, while in the flooded area, the numbers were 1,11 ind./m^2^, and the biomass 12,84 mg/m^2^. In the quantitative samples only Corixidae were found (*Sigara striata*, *Sigara stagnalis*, *Hesperocorixa linnaei* dominated), and during general collecting singular specimens of other families were found. In the extra inundated basins the quantitative characteristics were more than 10 times higher: the numbers were 19,68 ind./m^2^, the biomass was 220,79 mg/m^2^, dominated by *Sigara stagnalis*, *Sigara lateralis*, *Sigara assimilis* and *Hesperocorixa linnaei*. In quantitative samples besides Corixidae, there were also species of Pleidae, Gerridae, Notonectidae.

Throughout the next two weeks, the numbers and the biomass of water bugs in the riverbed and the flooded area continued to increase quickly. In the riverbed the numbers reached 3,02 ind./m^2^, the biomass - 36,19 mg/m^2^. In the flooded area the numbers went up 7 times (up to 7,58 ind./m^2^), and the biomass – almost 14 times (up to 167,07 mg/m^2^). The higher increase of the biomass in comparison with the numbers of water bugs was due to migration of larger species – *Notonecta viridis*, *Notonecta glauca*, *Ilycoris cimicoides*, to the flooded area, but the main contribution to the biomass and the numbers in these biotopes still belonged to Corixidae (6,16 ind./m^2^, 58,99 mg/m^2^). In the riverbed the four dominant Corixidae species were presented approximately in a peer ratio, in the flooded area *Sigara stagnalis* prevailed.

Closer to the middle of the spring period the majority of overwintered Gerridae species (*Gerris argentatus*, *Gerris odontogaster*, *Gerris thoracicus*) occupied not only the riverbed and the flooded areas but also the extra inundated basins, so the total numbers and biomass there increased a little, despite of migration of water bugs to the inundated water bodies. In the middle of April 2009, the numbers in the inundated basins reached 24,22 ind./m^2^, the biomass - 237,56 mg/m^2^, and Corixidae (*Sigara stagnalis*, *Sigara lateralis*) continued to dominate.

In the middle of the spring period (in April) the highest number and biomass per square meter and the greatest numbers of species were observed in the flooded area where mating of the wintered imagoes and oviposition took place. Up to the middle of April 2009, the numbers in the riverbed reached 6,67 ind./m^2^, the biomass - 169,44 mg/m^2^, while in the flooded area - 10 ind./m^2^, and 213,7 mg/m^2^ respectively. During this time in the flooded area, *Plea minutissima* and *Microvelia reticulata* appeared and started reproduction. In both types of inundated biotopes, Corixidae dominated by numbers (in the riverbed 3,7 ind./m^2^, in the basins 5,56 ind./m^2^), and Naucoridae - by biomass (74,81 and 130,74 mg/m^2^ respectively). The numbers and the biomass of water bugs in the extra inundated basins decreased by several times (to 5,4 ind./m^2^ and 47,62 mg/m^2^, respectively), by both indicators Corixidae dominated.

During **the late spring period** in the riverbed and the flooded area, the numbers continued to increase, and the biomass decreased, due to the appearance of nymphs and the death of overwintered imagoes. The first nymphs of Corixidae and Notonectidae appeared in flooded area in the middle of May. On 17.05.2009 the numbers were 11,39 ind./m^2^ in the riverbed, and 14,89 ind./m^2^ in the flooded area, the biomass – 61,39 and 52,79 mg/m^2^ respectively. The maximum numbers in the flooded area coincided with the minimum numbers in the extra inundated basins. In 2009 the minimum of the numbers and the biomass was 1,56 ind./m^2^ and 10,22 mg/m^2^ respectively. In the years with lower water levels (as for example 2008), the flooded area was smaller, they started to dry up at the end of April and therefore the difference in the numbers of water bugs between inundated and extra inundated basins was not so great.

During the late spring period, the highest numbers in the riverbed and the flooded area belonged to *Plea minutissima* imagoes (9,72 and 11,1 ind./m^2^ respectively), in the flooded area an essential contribution was also made by young nymphs of Notonectidae. In the extra inundated basins nymphs appeared later, therefore in the middle of May there were only single adult specimens, among which *Sigara stagnalis* and *Sigara lateralis* prevailed.

During this period the overwintered nymphs of Micronectidae became adults and started reproduction. They did not move to the flooded area, some imagoes moved to the riverbed, but generally they remained in the extra inundated basins. The numbers of Micronectidae in the basins in the middle of May were 52,78 ind./m^2^ and the biomass – 50 mg/m^2^.

At the end of the late spring period (the end of May – the beginning of June) nymphs of Naucoridae, Veliidae, Gerridae appeared, and up until the middle of June the first nymphs of Pleidae were noticed. After desiccation of the flooded area, the riverbed became an optimum habitat for reproduction and development of water bugs. Some adult specimens came back to the extra inundated basins. In the basins, nymphs appeared approximately two weeks later than in the flooded area and the riverbed, possibly due to the later heating of these biotopes. Most of the nymphs developed in the riverbed; therefore the quantitative characteristics there were much higher than in the extra inundated basins. The numbers were 34,4 ind./m^2^ in the basins, and 150 ind./m^2^ in the riverbed, the biomass – 170,4 and 498,89 mg/m^2^ respectively. The ratio between nymphs and imagoes was about 2,5:1 in the basins, and 14:1 in the riverbed. Young nymphs of Corixidae and Notonectidae dominated, but certain nymphs of small species of Corixidae (*Sigara stagnalis*, *Sigara lateralis*) and some Notonectidae (*Notonecta glauca*) in the riverbed had already managed to reach the fourth stage.

At the beginning of **the summer-autumn period** quantitative characteristics in the riverbed and in the extra inundated basins continued to increase. Biomass/numbers ratio in the riverbed was higher, than in the basins. It implies more favorable conditions for the development of the nymphs in the riverbed and so they matured earlier. At the end of June 2009, the numbers in the riverbed were lower than in the basins (223,33 and 340,83 ind./m^2^ respectively), while the biomass, on the contrary, was higher (3403,89and 914,44 mg/m^2^ respectively). The numbers of the nymphs in 1^st^-2^nd^ stages in the riverbed were almost 3 times lower, than in the basins (104,44 and 284,17 ind./m^2^ respectively), and the numbers of the nymphs in 4th-5th stages in the riverbed was 7 times higher (31,1 and 4,15 ind./m^2^ respectively).

The first nymphs of the new generation of Micronectidae also appeared in the riverbed and the extra inundated basins at the beginning of the summer-autumn period. At the beginning of June 2009 the numbers of Micronectidae nymphs were 25,19 ind./m^2^ in the basins, and 2,2 ind./m^2^ in the riverbed.

In June the riverbed began to dry. The adult water bugs moved from the drying riverbed to the extra inundated basins, as a result the numbers in the riverbed decreased. The biomass continued to be rather high, due to the high numbers of nymphs growing to 4th-5th stages and imago. Drying of the riverbed in 2009 started at the end of June - the beginning of July. In the middle of June 2009, the numbers in the riverbed were 52,78 ind./m^2^, and the biomass - 50 mg/m^2^. Dominant in numbers was the nymphs of *Plea minutissima* of elder stages, while dominant in biomass were the nymphs of elder stages and imago of *Notonecta glauca*, *Notonecta viridis*, *Ilyocoris cimicoides*, *Hesperocorixa linnaei*.

When the riverbed completely dries, water bugs gather in the extra inundated basins where the second reproduction of polivoltine species takes place. In 2009 the riverbed completely dried up at the end of July. The adult *Hesperocorixa linnaei* dominated in numbers and biomass. At the same time the first nymphs of the second generation of *Plea minutissima*, *Sigara stagnalis* and *Sigara lateralis* appeared.

In the middle of July, most nymphs of *Micronecta scholtzi* also finished their development, and separate individuals managed to couple and oviposite so that at the end of July the first nymphs of the next generation appeared. The second generation of polivoltine Corixidae was probably rather extended, because nymphs of younger stages were found until the beginning of November. The nymphs of Corixidae in 1^st^-2^nd^ stages dominated in numbers and biomass in September samples (20.09.2009, 88,9 ind./m^2^ and 115,56 mg/m^2^). At the beginning of October, nymphs of 4th-5th stages prevailed, but imagoes mostly belonging to *Sigara lateralis* had the highest total number. The highest biomass was due to the adult Notonectidae. Up until the end of October the majority of Corixidae turned into imago. At the end of October-November in the extra inundated basins there were a large numbers of species which gathered for wintering. High numbers were shown by *Sigara striata* (up to 57,22 ind./m^2^), *Sigara lateralis* (up to 67,2 ind./m^2^), and also *Sigara stagnalis* (up to 10,56 ind./m^2^), *Hesperocorixa linnaei* (up to 12,78 ind./m^2^), *Notonecta glauca* (up to 4,4 ind./m^2^) and *Notonecta viridis* (up to 6,67 ind./m^2^).

In the south of Ukraine, *Micronecta scholtzi* most likely had a partial third generation where the first nymphs appeared at the end of September. Nymphs of younger stages were observed up to the middle of November – most likely, the majority of them managed to reach the 4^th^ stage and safely overwintered. Imagoes were observed up to the beginning of November, after which there were only nymphs, and the wintering stages prevailed. The decrease in numbers at the beginning of November in comparison with October samples was probably due to them leaving the shoreline for wintering in the bottom remote parts of the basins.

Next settling of the riverbed depends on the period of its autumn filling. During a late filling, bugs don’t manage to occupy the riverbed and winter only in the constant extra inundated basins; during an early filling, a part of the bugs gathers for wintering in the riverbed, concentrating in creeks. It’s a risk, because during cold years the riverbed can freeze to the bottom, and water bugs trapped there die. On the other hand, because of the faster warming up of the riverbed in spring, the individuals wintering there can start the reproduction earlier.

Our study of seasonal changes in the numbers of Heteroptera imagoes and nymphs gives an opportunity to clarify some characteristics of the life cycle for the following mass species:

*Notonecta glauca* in the studied area usually has one generation per year. Mating is observed in March; first nymphs appear in the middle of May, imagoes of new generation – from the middle to the end of June. Therefore, most of *Notonecta glauca* nymphs grow in the flooded areas, and after their desiccation in the riverbed; then during the imago stage, they move to the extra inundated basins, where they dispause. This diapause is optional, and during the warm years *Notonecta glauca* can also have a partial second generation.

*Notonecta viridis* also has one generation per year, but the term of its development differs so the first imagoes of the new generation appear at the beginning of July. Therefore, not all the nymphs of *Notonecta viridis* have enough time to finish their development before the complete desiccation of the riverbed; that is why *Notonecta viridis* stays for reproduction in the extra inundated basins more often than *Notonecta glauca*,and their number in the extra inundated basins is higher than the number of *Notonecta glauca*.

*Hesperocorixa linnaei* in the studied area also has one generation per year. First imagoes of the new generation appear in June, reaching the maximum number in the middle of July. This fact fits with the literary data that another close species, *Hesperocorixa castanea*,is also univoltine (Saulich and Musolin 2007). In the literature for *Hesperocorixa castanea*,a partial second generation was reported, and most likely during the warm years, *Hesperocorixa linnaei* can also have a partial second generation.

*Sigara stagnalis* and *Sigara lateralis* belong to polivoltine species which have at least two generations per year in the region. Imagoes of the first generation appear from the middle to the end of June, imagoes of the second generation – in September. These facts fit with the literature data about the life cycle of *Sigara lateralis* in Rostov area (Russia) (Sokolskaya and Zhiteneva 1973) They may have a partial third generation, at least, in September we observed the nymphs of younger stages, but it is not clear if they belong to the third generation or to the late second generation. Maximum numbers of imagoes of these species was observed in October.

*Plea minutissima* is also polivoltine species which has at least two generations. Nymphs of the first generation appear from the beginning to the middle of June, in the middle of July they imaginate and begin to mate, and soon the first nymphs of the second generation appear. Almost all the first cycles of reproduction take place in the flooded areas and the riverbed, and the second in the extra inundated basins.

Reproduction of *Ilyocoris cimicoides* starts at the end of May. Oviposition is prolonged; therefore nymphs of different stages may be noticed simultaneously through the whole summer.

## Conclusions

In the studied rivers and associated water bodies we have found 28 species of water bugs belonging to 10 families and 15 genera. That is 67% of the total number of species registered in the steppe zone of the south Ukraine. This fact shows the important role of intermittent rivers in the forming of regional biodiversity of water bugs. Two species (*Sigara fossarum* and *Hydrometra gracilenta*) were registered for the first time in south Ukraine, and for *Micronecta scholtzi* this is the first proven record in the Ukraine.

Seasonal changes in species numbers, biomass and number of water bugs in the ecosystem of small intermittent rivers are undulating and depend on the climatic conditions of each year and peculiarities of life cycles of the dominant species. Among registered species there are polivoltine (*Sigara stagnalis*, *Sigara lateralis*, *Plea minutissima*) and univoltine (*Notonecta glauca*, *Notonecta viridis*, *Hesperocorixa linnaei*, *Ilyocoris cimicoides*) species, but some univoltine species may have a partial second generation. Almost all species overwinter as imago, excluding Micronectidae wintering at a nymphs’ stage, usually at the 4^th^ stage. Wintering at the egg stage was not registered.

During the early spring period, the maximum of quantitative indicators was observed in the extra inundated basins. During the middle of the spring period, maximum of the numbers, and later of the biomass was observed in the flooded area. During the late spring period and the beginning of the summer-autumn period, the maximum of quantitative indicators was observed in the riverbed. Maximums of the numbers (449 ind./m^2^) and the biomass (8811 mg/m^2^) were observed in the extra inundated basins in the middle of summer-autumn period (the end of July – the beginning of August) after desiccation of inundated biotopes and appearance of nymphs of the second generation of polivoltine species. Thus, during the droughty period when the riverbed is dry, the extra inundated basins (including artificial ones – sandy pits) serve as refugia for aquatic Heteroptera. The wintering of many species takes place there, especially when the riverbed is not filled before the cold period. However, the reproduction of the most species takes place in the riverbed and associated flooded area. Thus, this study is a confirmation and development of the concept for “a uniform architectonic complex of a river valley” (Beklemishev 1970).
